# Regulation of Osteoblast Differentiation and Iron Content in MC3T3-E1 Cells by Static Magnetic Field with Different Intensities

**DOI:** 10.1007/s12011-017-1161-5

**Published:** 2017-10-19

**Authors:** Jiancheng Yang, Jian Zhang, Chong Ding, Dandan Dong, Peng Shang

**Affiliations:** 1 Key Laboratory for Space Bioscience and Biotechnology, Institute of Special Environmental Biophysics, School of Life Sciences, Northwestern Polytechnical University, Xi’an, China; 2 School of Radiation Medicine and Protection, Collaborative Innovation Center of Radiation Medicine of Jiangsu Higher Education Institutions, Medical College of Soochow University, Suzhou, China; 3 Province-Ministry Joint Key Laboratory of Electromagnetic Field and Electrical Apparatus Reliability, School of Electrical Engineering, Hebei University of Technology, Tianjin, China; 4 Research and Development Institute in Shenzhen, Northwestern Polytechnical University, Shenzhen, China

**Keywords:** Static magnetic fields, Osteoblast, Differentiation, Iron

## Abstract

Many studies have indicated that static magnetic fields (SMFs) have positive effects on bone tissue, including bone formation and bone healing process. Evaluating the effects of SMFs on bone cell (especially osteoblast) function and exploring the mechanism, which is critical for understanding the possible risks or benefits from SMFs to the balance of bone remodeling. Iron and magnetic fields have the natural relationship, and iron is an essential element for normal bone metabolism. Iron overload or deficiency can cause severe bone disorders including osteoporosis. However, there are few reports regarding the role of iron in the regulation of bone formation under SMFs. In this study, hypomagnetic field (HyMF) of 500 nT, moderate SMF (MMF) of 0.2 T, and high SMF (HiMF) of 16 T were used to investigate how osteoblast (MC3T3-E1) responses to SMFs and iron metabolism of osteoblast under SMFs. The results showed that SMFs did not pose severe toxic effects on osteoblast growth. During cell proliferation, iron content of osteoblast MC3T3-E1 cells was decreased in HyMF, but was increased in MMF and HiMF after exposure for 48 h. Compared to untreated control (i.e., geomagnetic field, GMF), HyMF and MMF exerted deleterious effects on osteoblast differentiation by simultaneously retarding alkaline phosphatase (ALP) activity, mineralization and calcium deposition. However, when exposed to HiMF of 16 T, the differentiation potential showed the opposite tendency with enhanced mineralization. Iron level was increased in HyMF, constant in MMF and decreased in HiMF during cell differentiation. In addition, the mRNA expression of transferrin receptor 1 (TFR1) was promoted by HyMF but was inhibited by HiMF. At the same time, HiMF of 16 T and MMF of 0.2 T increased the expression of ferroportin 1 (FPN1). In conclusion, these results indicated that osteoblast differentiation can be regulated by altering the strength of the SMF, and iron is possibly involved in this process.

## Introduction

All the organisms on the Earth are continuously exposed to intrinsic geomagnetic field (GMF, 25-65 μT), which plays an essential role in living. Besides GMF, chances for human exposed to various static magnetic fields (SMF) have increased a lot with rapid development in science and technology, such as magnetic resonance imaging (MRI), overhead cables with high-voltage direct current, and some transportation systems based on magnetic levitation. Furthermore, the intensity of SMF in deep space for astronaut is much lower than the GMF: < 300 nT on the moon and ~ 6 nT in interplanetary space. Growing evidence suggests that deprivation of GMF (i.e., hypomagnetic field, HyMF) has adverse impacts on many functional states of organisms (reviewed in [1]). Thus, studying the biological effects under HyMF can help not only better understand the GMF’s function on the health of human beings, but also predict the potential effects of HyMF on the health of astronaut during interplanetary navigation. Under high SMF (HiMF) of up to several teslas, vertigo, nausea, and phosphenes may occur in some people due to peripheral nerve stimulation and perturbation of the vestibular system. Nevertheless, there is no convincing evidence that moderate SMF (MMF) or HiMF would induce any adverse effects [2]. Certain SMFs are also used to keep healthy or treat some diseases nowadays [3]. Therefore, it is necessary to systematically elucidate the biological effects and mechanisms of SMFs ranging from hypomagnetic field (HyMF, < 5 μT), weak SMF (WMF, 5 μT–1 mT), moderate (MMF, 1 mT–1 T) to high (HiMF, > 1 T).

Many animal studies concerned with health effects have demonstrated that SMFs with moderate intensity can improve bone formation with increased bone mineral density (BMD) and enhance bone healing in numerous circumstances, such as bone surgical invasion [4], ischemic bones [5], adjuvant arthritis rats [6], bone fracture [7], ovariectomized rats [8], and bone grafts [9]. HyMF aggravates bone loss induced by hindlimb unloading in rat femurs [10]. Most studies believe that these positive impacts on bone are related to the function enhancement of osteoblast [11]. Osteoblasts arise from mesenchymal stem cells and function as bone synthesis and mineralization. At cellular level, SMF do have some modulations on behaviors and function [12], such as morphology, proliferation, cell cycle distribution, apoptosis, differentiation, gene expression etc. Previous studies demonstrate that SMFs especially MMF promote osteoblast differentiation. The ability of osteogenic differentiation in various osteoblastic cells is enhanced under moderate SMF, including human osteosarcoma cell lines MG63 [13–15], mouse calvarial osteoblast MC3T3-E1 [16–18], and rat calvaria cells [19]. It should be noted that almost all the researches focus on MMF generated by permanent magnets due to its easy realization. Moreover, the experimental design, exposure facility, magnetic induction (ranging from HyMF to HiMF), and types of biological samples used (animals, cells, or molecules) are largely heterogeneous. Thus it is difficult to draw a definite conclusion that how bone cells respond to SMF.

Iron is essential for almost all living organisms and is crucial for many biological processes such as the oxygen transport and enzymatic reactions [20]. In recent years, preclinical and clinical studies have demonstrated a close relationship in iron metabolism and bone metabolism [21]. Iron overload or iron deficiency can cause abnormal bone metabolism or osteoporosis. Excess iron that could inhibit the biological activity of osteoblasts has been demonstrated in vitro experiments [22–24]. Low iron, in contrast, inhibits osteoblastogenesis in vitro as well [24]. Although iron and magnetic fields have the natural relationship, there are few studies concerning the effect of SMFs on iron at the level of biochemistry. Recently, a study showed that SMF exposure with 128 mT alters the plasma levels of iron in rats [25].

In order to comprehensively examine the regulatory role of SMF on osteoblast, and whether iron involve in the altered bone formation by osteoblast under SMFs, the present study was undertaken to investigate differentiation and iron changes of osteoblastic MC3T3-E1 cells under such SMFs range from HyMF, GMF, MMF to HiMF. In this study, three types of SMF exposure systems were used according to our previous research [26, 27]. HyMF of about 500 nT (created by magnetic shielding room), MMF of 0.2–0.4 T (surrounding a superconducting magnet), and HiMF of 16 T (generated by a superconducting magnet) were employed to simultaneously investigate the SMF effects.

## Materials and Methods

### The Facilities of SMFs Exposure for Cell Culture

**Fig. 1:**
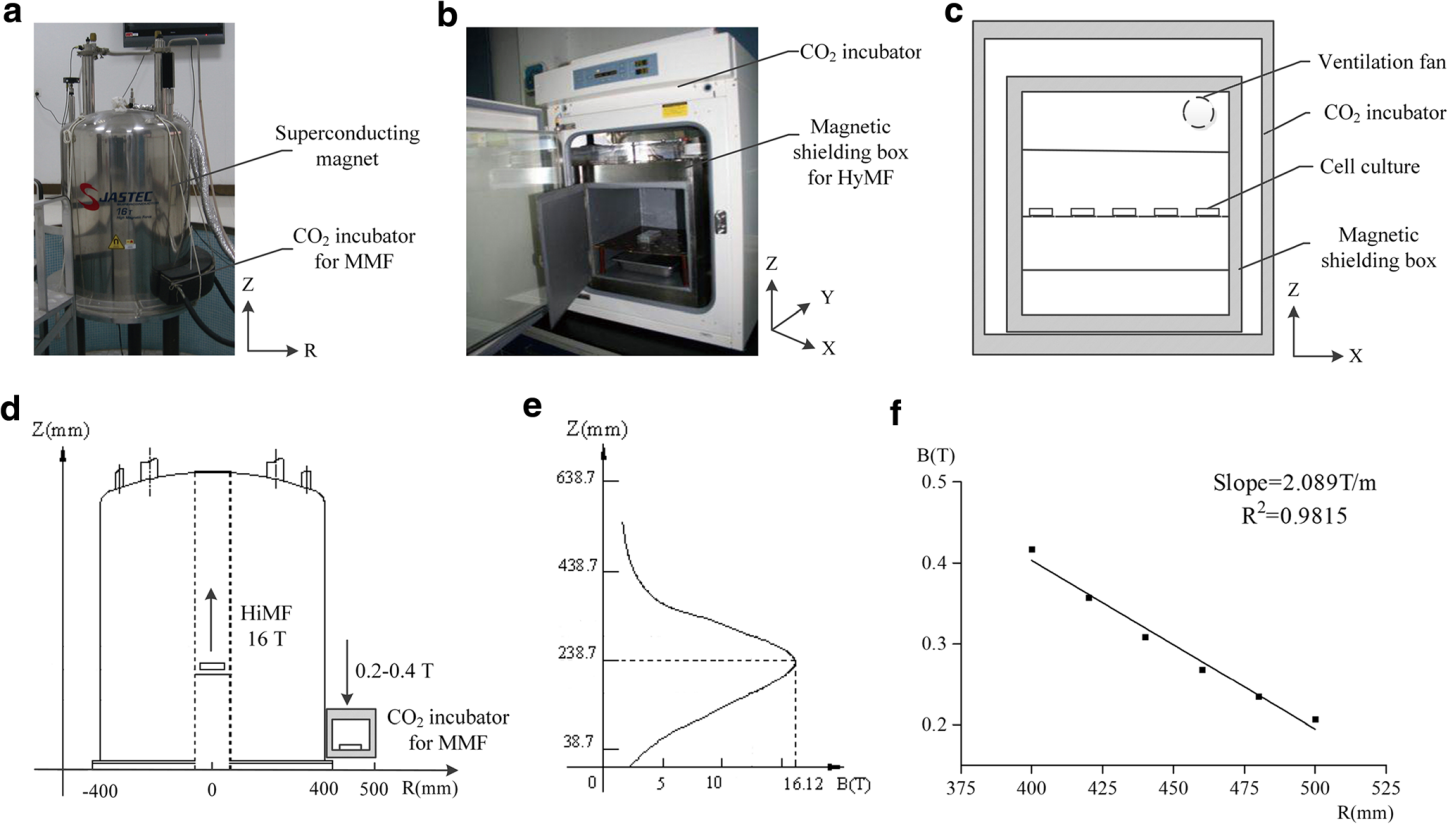
Schematic diagram of the SMF exposure systems for cell culture. In this study, HiMF and MMF were produced by a vertical cylindrical-type superconducting magnet (**a**). The side view of the magnet (**d**) was shown to illustrate the position of cell culture, where arrow represented magnetic field direction. **e** and **f** showed the distribution of magnetic field at the center bore of the magnet along Z axis and in the circular area around the magnet, respectively. A permalloy magnetic shielding box designed for the realization of HyMF was placed in a CO_2_ incubator (**b**). As the side view of the magnetic shielding box (**c**) shown, a fan was installed on the top to facilitate the gas and heat exchange with the cell culture incubator. *B* magnetic flux density, *T* tesla, *R* radius from center of the superconducting magnet

HiMF of 16 T was generated by a superconducting magnet (JASTEC, Kobe, Japan), and the cell culture was maintained in the central bore of the magnet (Fig. 1a and d). The distribution of the magnetic field along the Z axis of 238.7 mm was the highest, and its intensity was about 16 T (Fig. 1e). MMF of 0.2 T was achieved in a circular space around the superconducting magnet, where the distribution of magnetic field was about 0.2–0.4 T with a decreased gradient of 2 T/m along the radium direction (Fig. 1d and f). For cell culture, we have established an experimental platform for biological research in and around the superconducting magnet as we described [28–30]. The temperature was kept at 37 °C by heating circulating water baths, and the concentration of CO_2_ was 5% calibrated by a CO_2_ analyzer (Geotech, Leamington, UK).

HyMF was achieved by magnetic shielding technology [10]. A magnetic shielding box (550 mm × 420 mm × 420 mm) made of permeability alloy (NORINDAR International, Shijiazhuang, Hebei, China) was used to create a hypomagnetic condition, where the magnetic field strength was approximately 500 nT (Fig. 1b and c). The shield box was put in a cell incubator (Thermo Fisher Scientific, Waltham, MA, USA) and a fan installed to ensure the optimal conditions of cell culture (5% CO_2_, 37 °C).

Cells of GMF control were cultured in a normal cell incubator (Thermo Fisher Scientific) where the magnetic field was about 45 μT and slightly lower than the local GMF in the laboratory (~ 55 μT) due to the magnetic shielding effect of the incubator. The intensity of magnetic field was measured by a gaussmeter (Lake Shore Cryotronics, Westerville, OH, USA). The alternative current (AC) magnetic fields generated by the incubator and the fans of the magnetic shielding box were measured previously [31]. The AC field in the GMF control incubator and magnetic shielding chamber was 1013.2 ± 157.5 nT and 12.0 ± 0.0 nT, respectively, which was much smaller than the intensity of GMF. Besides, the predominant frequency was 50 Hz, equal to the used power line frequency. The temperature and CO_2_ were set at 37C^o^ and 5%, respectively, to ensure the optimal conditions of cell culture.

### Cell Culture

Murine osteoblastic cell line MC3T3-E1 Subclone 4 [32] was used in this study and kindly provided by Prof. and Dr. Hong Zhou of the University of Sydney. The osteoblastic MC3T3-E1 cells were maintained by *α*-Minimum Essential Medium (*α*-MEM; Gibco, Grand Island, NY, USA), supplemented with 2 mM L-glutamine, 10% (*v*/*v*) fetal bovine serum (FBS; Gibco) in a humidified 5% CO_2_ atmosphere at 37 °C.

### Hematoxylin-Eosin Staining

Cell morphology was monitored by hematoxylin-eosin (HE; Beyotime, Shanghai, China) staining. The cells were seeded on coverslips and pre-cultured for 24 h at a density of 3000 cells/cm^2^ and then continuously exposed to SMF for 2 days. After that, cells were fixed by 4% paraformaldehyde, and then stained by 0.5% hematoxylin for 7 min and 0.5% eosin for 7 min. Digital images were obtained by using a Nikon Eclipse 80i microscope (Nikon, Tokyo, Japan). For statistical analysis, we selected 100 cells per group to quantify cell area and diameter of MC3T3-E1 cells by Image J software (National Institutes of Health, USA; http://imagej.nih.gov/ij/).

### Cell Proliferation Assay

The cells (8000 cells/cm^2^) were planted in 96-well plates (Corning, NY, USA). The proliferation of MC3T3-E1 cells was measured by MTT assay. Briefly, osteoblasts were uninterruptedly cultured in SMFs for 48 h; thereafter, MTT dye solution was added. Continue to incubate for 4 h, the supernatant was removed and DMSO was added to solubilize the MTT. The absorbance was read at 570 nm using a microplate reader (Bio-Rad Laboratories, Hercules, CA, USA).

### Cell Cycle Distribution Assay

MC3T3-E1 cells were first seeded at 3000 cells/cm^2^ in petri dishes with 35 mm diameter and pre-cultured for 24 h. After that, the cells were synchronized at G0/G1-phase by serum starvation (*α*-MEM with 1% FBS) for 24 h. Then, the cells were transferred into normal medium and released in SMFs for 24 h. For cell cycle analysis, cells were washed with ice-cold phosphate buffered saline (PBS), fixed in 75% ice-cold ethanol overnight and stained by 50 μg/ml propidium iodide (PI; Sigma-Aldrich) and 1 mg/ml RNase A (Sigma-Aldrich) for 60 min. Cell cycle was detected and analyzed with a flow cytometer (BD Bioscience, Franklin Lakes, NJ, USA).

### Mineralization Assay

The MC3T3-E1 cells (5 × 10^4^ cells/cm^2^) were seeded into 35 mm petri dishes. At confluence, osteogenesis by osteoblast MC3T3-E1 was induced by cell culture medium with ascorbic acid (50 μg/ml; Sigma-Aldrich) and *β*-glycerophosphate disodium salt hydrate (10 mM; Sigma-Aldrich). The cells were then constantly exposed to SMF for day 8, and osteogenic media was changed every 48 h. For mineralization assay, mineralized osteoblast cultures were fixed in 4% paraformaldehyde and then stained by 0.1% Alizarin red S (Sigma-Aldrich). Positive alizarin red staining for calcium represented the calcium phosphate of osteoblast culture mineralization. Alizarin red-stained osteoblast cultures were photographed by a scanner, and the total area of red calcified nodules was measured by Image J software (National Institutes of Health).

### ALP Activity Assay

The MC3T3-E1 cells (5 × 10^4^ cells/cm^2^) were seeded into 96-well plates. At confluence, osteogenic medium was used and changed every 48 h. After uninterrupted treat with SMF, cells were harvested at certain time points (from 2 to 8 days with a 2-day interval). Intracellular ALP activity was evaluated by using *p*-nitrophenyl phosphate (*p*NPP; Sigma-Aldrich) assay based on the ability of phosphatases to hydrolyze *p*NPP to *p*-nitrophenol (*p*NP), a yellow soluble product under alkaline conditions with absorbance at 405 nm. Cells were washed twice with PBS and then lysed by three repeated freeze-thaw which cells were placed at − 80 °C and room temperature for 15-min intervals. One hundred and fifty microliter of *p*NPP was added into each well and incubated at 37 °C for 30 min. Absorbance was read at 405 nm. ALP activity was expressed as nanomole of *p*NPP hydrolyzed per 30 min per well (Corning, Tewksbury, MA, USA). Total protein was measured with a BCA kit (Thermo Fisher Scientific).

### Calcium Deposition and Iron Content Assay

The calcium deposition and iron content in osteoblast culture were determined by atomic absorption spectrometry (AAS; Analytik Jena, Jena, Germany) as previously described [26]. At mineralization period, cell culture was washed with 0.9% NaCl and then dissolved in 1 ml 65% HNO_3_ at 60 °C for 2 h. The dried samples were dissolved in 10 ml 0.1% HNO_3_. Calcium content in cells and iron content in cultural supernatant was detected by flame AAS, while iron content in cells was determined by graphite furnace AAS.

### qPCR

After 2 days of mineralization, total RNA was extracted with the TRIzol reagent (Invitrogen, Carlsbad, CA, USA) according to the manufacturer’s protocol and was used to synthesize cDNA with PrimeScript™ RT regent kit (TaKaRa, Liaoning, China). Then mRNA expression levels of the following genes were analyzed with quantitative real-time polymerase chain reaction (qPCR) assays and performed on CFX96 Touch qPCR System (Bio-Rad Laboratories, Hercules, CA, USA) using SYBR Premix Ex Taq™ (TaKaRa). The specific pairs of primers were listed in Table 1. The data were calibrated to GAPDH and analyzed via 2^−ΔΔCt^ method.

**Table 1:** Primer sequences used for quantitative real-time PCR

Gene name (Genebank No.)	Primer sequences (5′–3′)	Annealing temperature (°C)
GAPDH (NM_008084.2)	Forward: TGCACCACCAACTGCTTAG	55
	Reverse: GGATGCAGGGATGATGTTC	
ALP (NM_007431.1)	Forward: GTTGCCAAGCTGGGAAGAACAC	55
	Reverse: CCCACCCCGCTATTCCAAAC	
BSP (NM_008318.3)	Forward: AAAGTGAAGGAAAGCGACGA	55
	Reverse: GTTCCTTCTGCACCTGCTTC	
Col Iα1 (NM_007742.3)	Forward: GAAGGCAACAGTCGATTCACC	55
	Reverse: GACTGTCTTGCCCCAAGTTCC	
OC (NM_001032298.2)	Forward: GAACAGACTCCGGCGCTA	55
	Reverse: AGGGAGGATCAAGTCCCG	
DMP1 (NM_016779.2)	Forward: AGTGAGGAGGACAGCCTGAA	60
	Reverse: GAGGCTCTCGTTGGACTCAC	
OPN (NM_001204203.1)	Forward: TTCACTCCAATCGTCCCTAC	55
	Reverse: TGCCCTTTCCGTTGTTGTC	
TfR1 (NM_011638.4)	Forward: GATCAAGCCAGATCAGCATTCT	60
	Reverse: ACCGGGTGTATGACAATGGTT	
FPN1 (NM_016917.2)	Forward: ACCAAGGCAAGAGATCAAACC	60
	Reverse: AGACACTGCAAAGTGCCACAT	
H-ferritin (NM_010239.2)	Forward: CAAGTGCGCCAGAACTACCA	60
	Reverse: GCCACATCATCTCGGTCAAAA	
L-ferritin (NM_010240.2)	Forward: CCATCTGACCAACCTCCGC	60
	Reverse: CGCTCAAAGAGATACTCGCC	

The specific pairs of primers

### Statistical Analysis

All experiments were performed at least in triplicate. Summary data were reported as mean ± SD, compiled, and analyzed by Graph Pad Prism software (GraphPad, La Jolla, CA, USA). Mean ± standard deviation (SD) was calculated for each group using the appropriate version of one-way analysis of variance (ANOVA) with Newman-Keuls. *P* < 0.05 was considered statistically significant.

## Results

### Effects of SMFs on the Growth of Osteoblastic MC3T3-E1 Cells

**Fig. 2:**
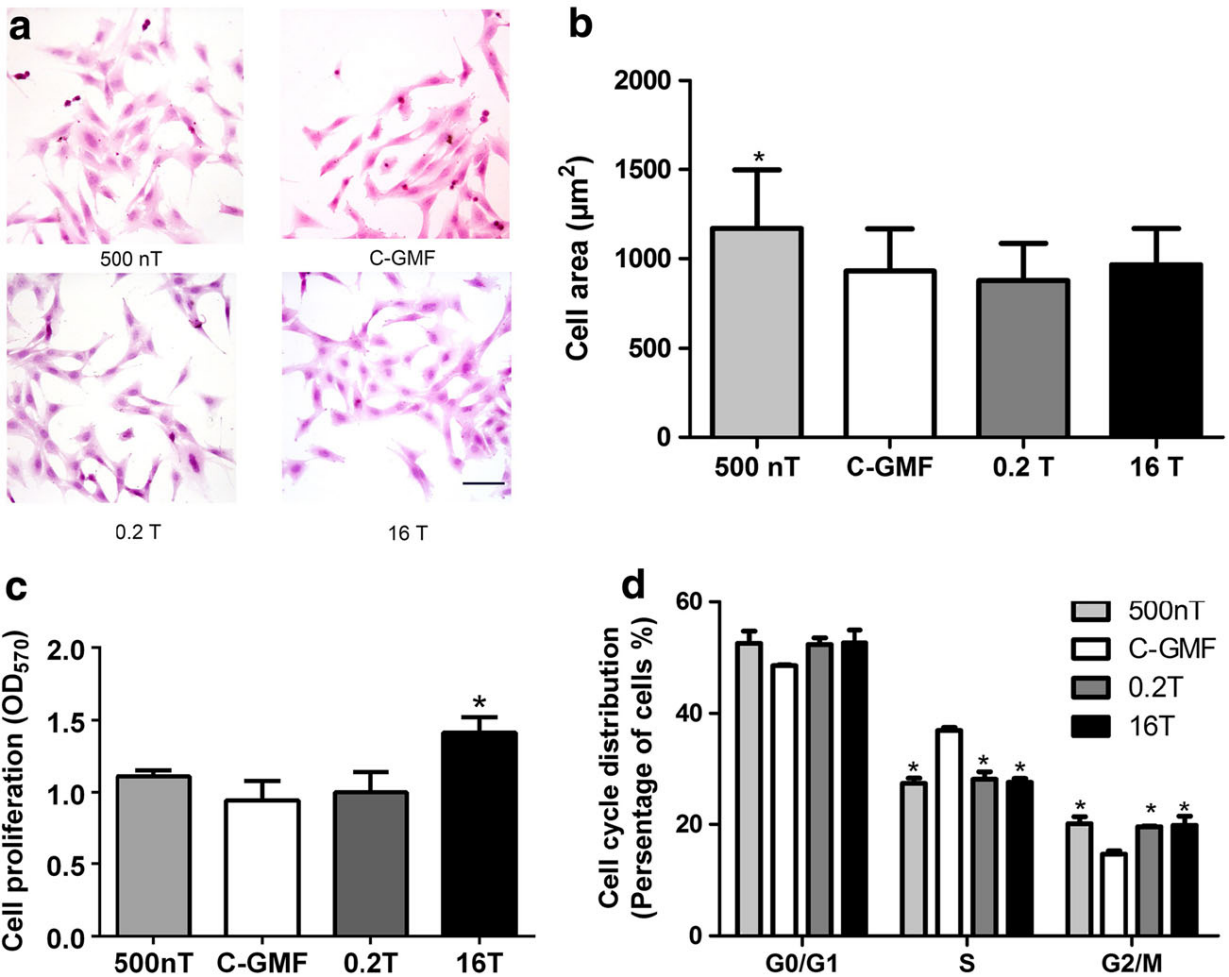
The cell growth of MC3T3-E1 cells under SMFs. **a** Cell morphology was measured by HE staining. Bar, 100 μm. **b** Cell area was analyzed by Image J software (*n* = 100). **c** The proliferation of MC3T3-E1 cells was examined by MTT assay, and the results were shown as optical density at 570 nm (OD_570_) (*n* = 3). **d** The cell cycle distribution of MC3T3-E1 cells under SMFs. MC3T3-E1 cells were synchronized at G0/G1-phase by serum starvation and then released under varied SMFs for 1 d. Cell cycle distruibution was determined by flow cytometry with PI staining (*n* = 3). All SMF groups were compared with the GMF of 0.05 mT group. Data shown are mean ± SD, *P < 0.05

In the present study, we first evaluated whether or not the osteoblastic MC3T3-E1 cells could survive and grow well under SMF. The MC3T3-E1 morphology was examined. After SMFs treatment for 2 days, MC3T3-E1 cells were not detached or became thinner apparently (Fig. 2a). However, when grown under 500 nT, MC3T3-E1 showed an increase in spread area (Fig. 2b).

In order to further investigate whether cells can grow well in these extreme man-made environments, cell proliferation was evaluated. After exposed to 16 T for 48 h, the proliferation of MC3T3-E1 cells was distinctly accelerated compared with that cultured under GMF, while the proliferative of cells exposed to HyMF and MMF for the same times was not significantly changed (Fig. 2c). This indicated that MC3T3-E1 cells could grow well regardless of whether they were treated with SMF including HyMF, MMF, and HiMF or not.

To verify whether SMF effects on cell proliferation was associated with cell cycle distribution, we tested whether or not SMF could alter the cell cycle distribution of MC3T3-E1 using flow cytometry. After MC3T3-E1, cells were synchronized at G0/G1-phase by serum starvation and then released with normal medium under SMFs for 1 day; SMF caused an increase in the proportion of G2/M-phase cells and a significant decrease in S-phase cells (Fig. 2d). These results may in part account for the stimulus effect on cell proliferation.

### Effects of SMFs on Iron Level of Osteoblastic MC3T3-E1 Cells During Proliferation

**Fig. 3:**
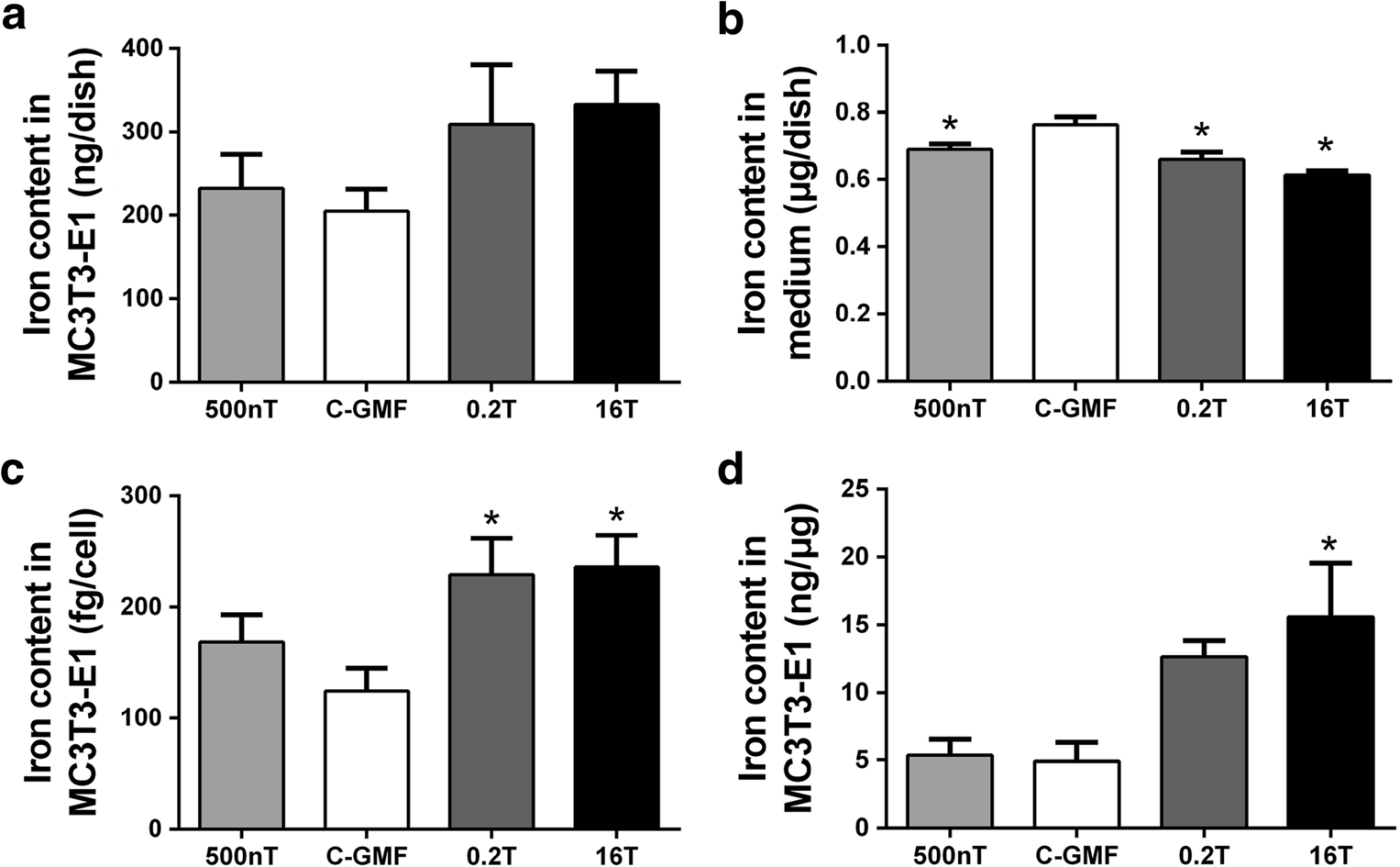
Effects of SMFs on iron level of osteoblastic MC3T3-E1 cells during proliferation. Iron level in cells was detected by graphite furnace AAS (**a**), and in culture medium was detected by flame AAS (**b**) and expressed as milligram per dish. The total iron content of each dish was normalized by the number of cells (**c**) and the total protein content per dish (**d**). All SMF groups were compared with the GMF of 0.05 mT group. Data shown are mean ± SD, *P < 0.05

During cell proliferation, osteoblastic MC3T3-E1 cells were exposed to SMFs for 48 h; there was not a significant change in total iron content of cell culture dishes (Fig. 3a), but the level of iron in cell culture medium was decreased significantly under SMFs (Fig. 3b). In order to eliminate the effect of cell inconsistent proliferation under SMFs, we normalized the total iron content with the number of cells and the total protein content per dish. The results indicated that elemental iron in each cell was increased under MMF of 0.2 T and HiMF of 16 T, but was not significant alterations in HyMF of 500 nT (Fig. 3c). Similarly, iron content per unit protein was elevated in MMF and HiMF, but did not show any changes in HyMF (Fig. 3d).

### Effects of SMFs on the Formation of Mineralization in Osteoblastic MC3T3-E1 Cells

**Fig. 4:**
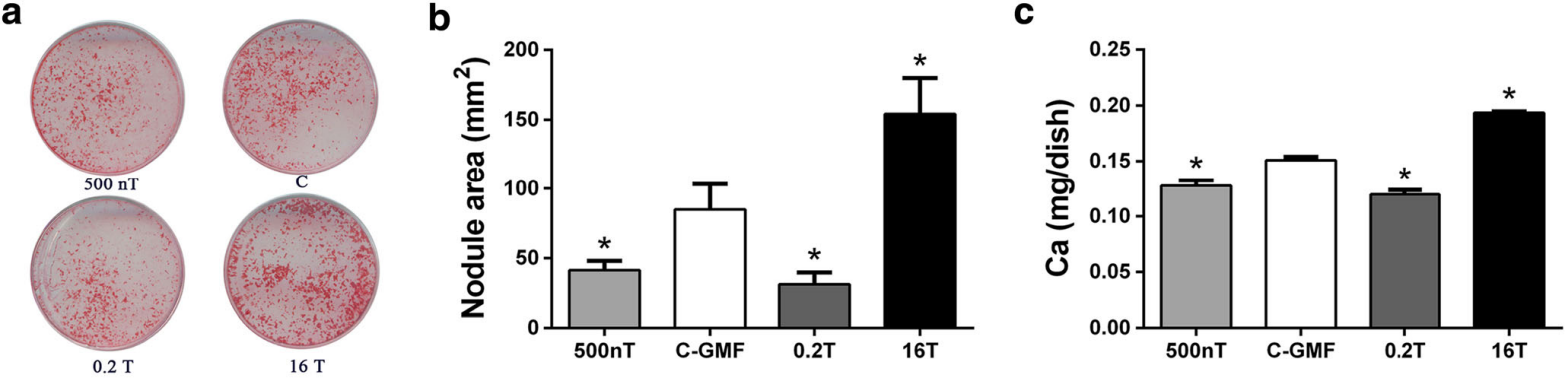
Effects of SMFs on mineralization process of osteoblastic MC3T3-E1 cells. Osteogenic differentiation was confirmed by alizarin red S staining (**a**) and analyzed by nodule area per dish (Diameter, 35 mm) at day 8 (**b**). Calcium deposition during mineralization was detected by flame atomic absorption spectrometry and expressed as milligram per dish at day 8 (**c**) *n* = 3. All SMF groups were compared with the GMF of 0.05 mT group. Data shown are mean ± SD, *P < 0.05

Osteogenesis from osteoblast is a complex process that involves three stages: cell proliferation, matrix maturation, and matrix mineralization [32]. When matrix maturation occurs, there is extensive expression of ALP and several matrix proteins, including BSP, Col, DMP1, OC, OPN, etc. Then, minerals mainly in the form of hydroxyapatite crystals are deposited in the matrix. The effects of SMFs on matrix mineralization of MC3T3-E1 cells were examined after continuous exposure 8 days. Matrix mineralization was characterized by analyzing the formation of calcified nodules. As shown in Fig. 3a, for the osteoblasts treated for 8 days, the area of formed nodules in 16 T group was significantly more than that of control group. Meanwhile, the formed nodules in 500 nT and 0.2 T were less than in GMF (Fig. 4a and b). In order to further evaluate the degree of mineralization, atomic absorption spectrometry was utilized to precisely analyze the calcium content in the mineralized cultures. Calcium deposition was enhanced by HiMF and declined by HyMF and MMF at day 8 (Fig. 4c).

### Effects of SMFs on ALP Activity of Osteoblastic MC3T3-E1 Cells

**Fig. 5:**
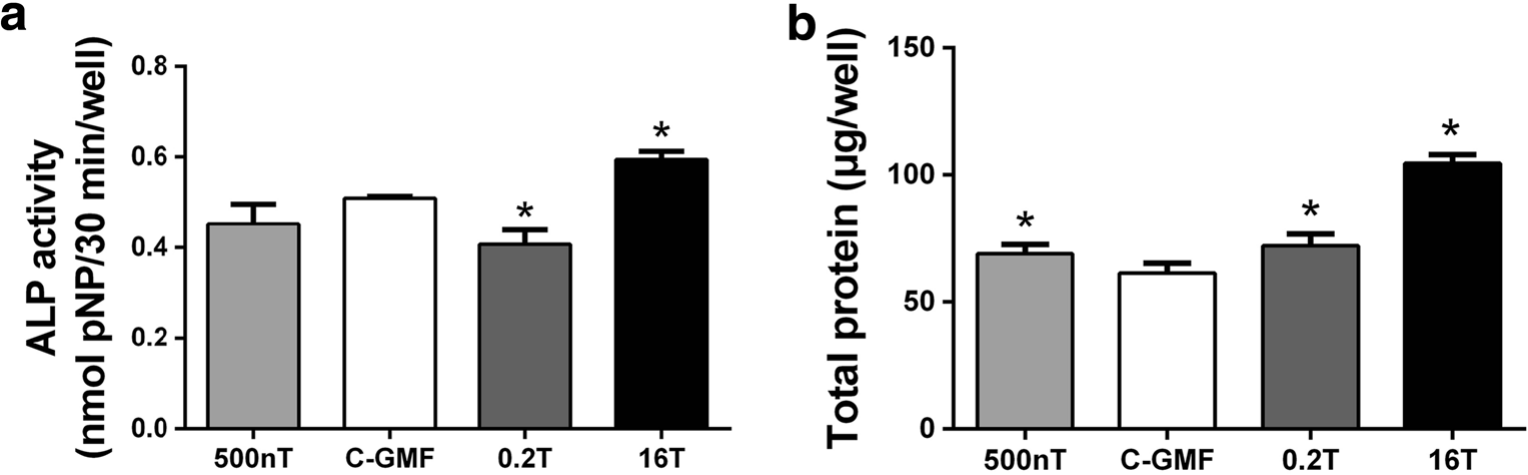
Effects of SMFs on ALP activity during osteoblast differentiation. **a** ALP activity in MC3T3-E1 cultures was detected by *p*NPP method at 8 days and expressed as micromoles of *p*NPP hydrolyzed per 30 min per well. **b** During differentiation, total protein was measured by BCA kit and expressed as microgram per well, *n* = 3. All SMF groups were compared with the GMF of 0.05 mT group. Data shown are mean ± SD, *P < 0.05

Mineralization is accompanied by increased activity and expression of ALP, which is regarded as markers during osteoblast differentiation. Consistently, the alteration of ALP activity was in a similar tendency as matrix mineralization. Compared to the control of GMF, treatments with 16 T significantly increased the ALP activity at day 8 (Fig. 5a). Moreover, total protein during osteoblast differentiation was significantly higher than GMF-control (Fig. 5b). The results indicate that the promotion of ALP activity under HiMF of 16 T may be associated with enhanced expression and secretion of ALP.

### Effects of SMFs on mRNA Expressions of Matrix Proteins

**Fig. 6:**
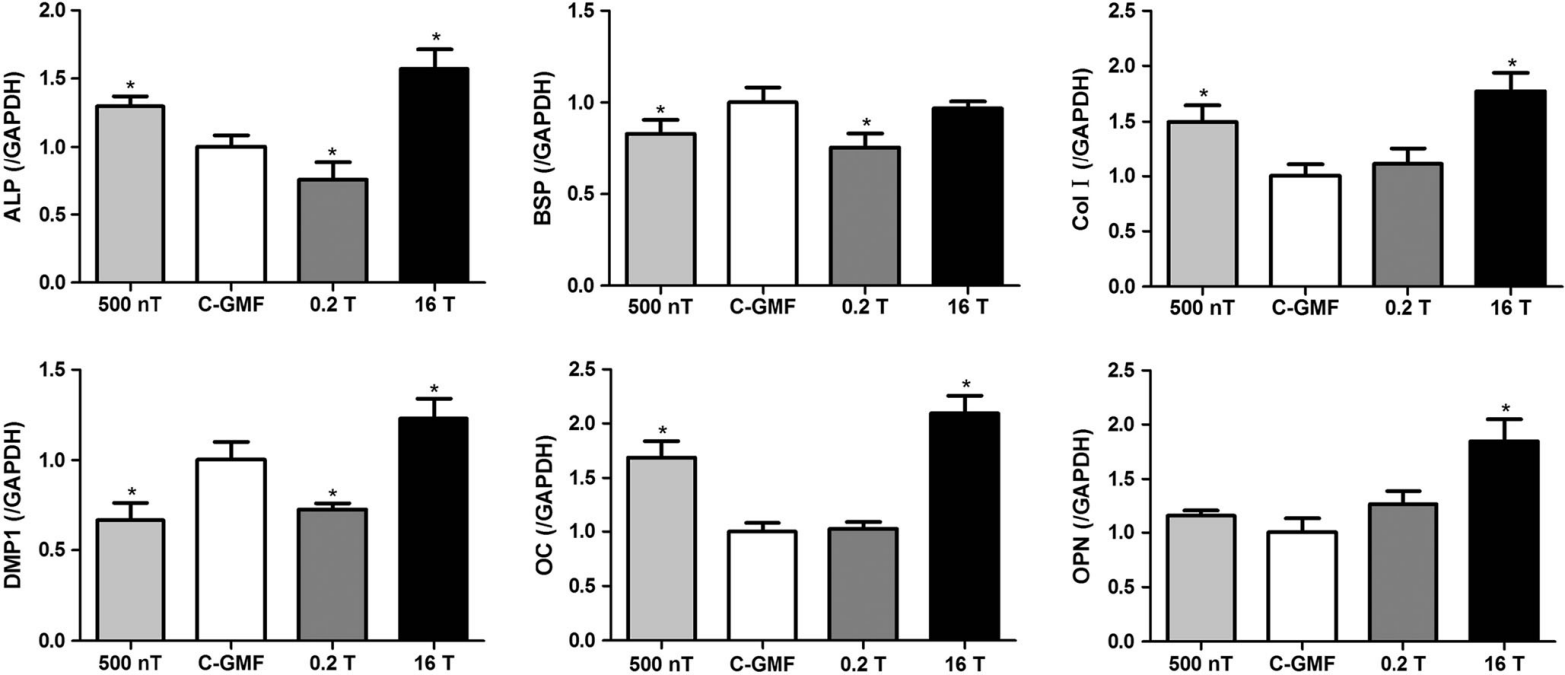
mRNA expressions of osteoblast matrix proteins during differentiation were measured by Q-RT-PCR after SMFs treatment for 2 days. All SMF groups were compared with the GMF of 0.05 mT group. Data shown are mean ± SD, *P < 0.05

During differentiation, osteoblast needs to synthesize and secrete bone matrix proteins including BSP, Col I, OC, OPN, DMP1 etc. In this study, total proteins were determined to represent the expression of matrix proteins during osteoblast differentiation. These osteogenic gene markers were detected based on polymerase chain reaction. Sixteen tesla exposure at day 8 significantly elevated the contents of total protein (Fig. 5b), which may be related to increased expressions of these bone matrix proteins after a 2-day stimulus of HiMF exposure (Fig. 6). The mRNA expression of ALP, BSP, and DMP1 was suppressed in MC3T3-E1 cells treated under 0.2 T. Moreover, cells from 500 nT group expressed less BSP and DMP1, but higher Col I and OC than that of control group.

### Effects of SMFs on Iron Metabolism of Osteoblasts During Differentiation

**Fig. 7:**
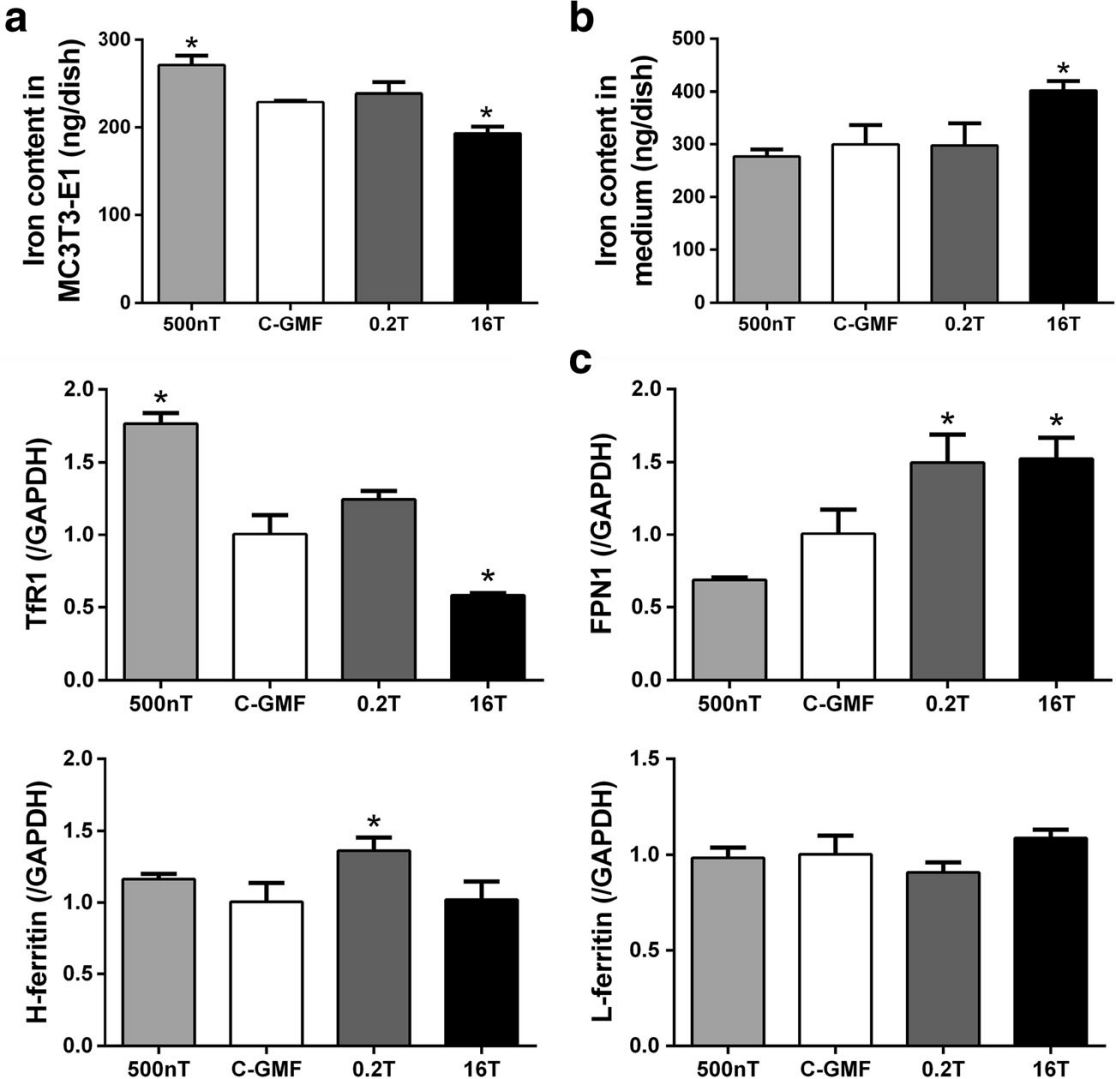
Effects of SMFs on iron metabolism of osteoblasts during differentiation. Iron content in cells was detected by graphite furnace AAS (**a**), and in culture medium was detected by flame AAS (**b**), and expressed as milligram per dish. mRNA expressions of iron metabolism markers in cells were measured by Q-RT-PCR 2 days after SMFs treatment (**c**). All SMF groups were compared with the GMF of 0.05 mT group. Data shown are mean ± SD, *P < 0.05

During mineralization, HyMF of 500 nT increased the level of iron in cells (Fig. 7a) and slightly reduced iron content in mediums (Fig. 7b). In contrast, the level of iron was decreased in cells and elevated in cultural supernatant under HiMF of 16 T. However, there is no significant changes on iron content in cells and mediums under 0.2 T (Fig. 7).

Under physiological conditions, iron in circulation is generally bound to transferrin (Tf). The uptake of Tf-bound iron through the membrane-bound transferrin receptor 1 (TFR1) is the main source of iron for most cells [33]. Once inside the cell, iron can be utilized and stored. Intracellular iron can be stored in ferritin; ferritins are composed of 24 similar subunits of two types, H and L. The H-subunit (H-ferritin) is responsible for the rapid oxidation of ferrous to ferric iron at a dinuclear center, whereas the L-subunit (L-ferritin) appears to help iron clearance from the ferroxidase center of the H-subunit and support iron nucleation and mineralization [34]. During iron egress, iron is exported by ferroportin 1 (FPN1) which is currently the only iron exporter to be identified in mammals [35]. In this experiment, the mRNA expression of some related genes in celluar iron metabolism was assessed, including TFR1, H-ferritin, L-ferritin, and FPN1 (Fig. 7c). The mRNA expression of TFR1 was promoted in MC3T3-E1 cells treated under 500 nT, but was suppressed under 16 T. Conversely, HiMF of 16 T and MMF of 0.2 T increased the expression of FPN1, while indistinctively decreased under 500 nT. There was an increased expression of H-ferritin under 0.2 T. Taken together, these results at the mRNA level indicated the inhibitory effect of 16 T on celluar iron uptake and the facilitative effect of 16 T on celluar iron efflux compared with that of the groups of control. However, there were a promoting effect on iron acquisition under 500 nT and iron excretion under 0.2 T.

## Discussion

To date, SMF has received increased attention as exposure from many different sources happens in various situations and its possible health effects have been studied in many fields such as cognitive systems, cardiovascular system, immune system, skeleton system etc. In the present study, we have assessed the influence of a board range of SMF from HyMF, GMF, MMF to HiMF on osteoblast differentiation and tried to understand the potential mechanism via iron metabolism for the first time. We found that such extreme SMF environments as GMF deprivation or HiMF did not have lethal effects on osteoblast viability. Osteoblast differentiation as well as celluar iron uptake and iron efflux can be controlled by altering the parameters of SMF, such as magnetic flux intensity.

We first examined whether or not MC3T3-E1 cells could survive well under such a board intensities of SMF by means of morphology, proliferation, and cell cycle distribution. Cell morphology demonstrated that SMFs did not result in distinct modifications of cell shape, but osteoblasts showed an increase in spread area under HyMF. However, a recent study showed that HyMF inhibited cell adhesion in human neuroblastoma cells, and cells were smaller in size and more round in shape [36]. These seemingly contradictory findings can be attributed to different cell types.

In this study, the results indicated that the effect of SMFs on cell proliferation differed in their characteristic responses to different magnetic intensities. Numerous studies have investigated the SMF effects on proliferation in various osteoblastic cell lines; these results are controversial. Our previous studies exhibit that 16 T promotes osteoblast proliferation in MC3T3-E1 and MG63 cells [37, 38]. But exposure to HiMF of 8 T exerts no effects on proliferation of MC3T3-E1 cells [18]. On the other hand, proliferation rate of MC3T3-E1 cells was decreased after treated with MMF of 250 mT [17]. Results from Huang et al. [14] indicate that the effects of SMF on osteoblast proliferation are associated with the initial cell densities. Cell-cell contact may influence the degree of reorientation and deformation of lipid bilayer or proteins embedded on the membrane by SMF. As the SMF affected cell proliferation, we monitored the cell cycle progression of MC3T3-E1 cell throughout the incubation period. 500 nT, 0.2 T, and 16 T not only decreased the percentage of S-phase cells, but also increased the number of G2/M-phase cells, indicating that transition through the DNA synthesis S phase to enter into the mitosis G2/M-phase. These results suggest that altered proliferation in osteoblast may be due to S-to-G2/M transition in cell cycle. Together, these published reports, with our findings, suggest that SMF do not have lethal effects on the viability of osteoblast from HyMF, MMF to HiMF.

Iron is essential for cellular growth and crucial to many fundamental cellular processes, including DNA synthesis, respiration, cell cycle regulation, and the function of proteins [33]. In this study, we observed the effects of SMF on cell proliferation with changed iron level. For under 16 T, osteoblast proliferation and elemental iron of each cell were dramatically promoted at 48 h. Therefore, it is potentially that increased elemental iron promotes cell proliferation. For under MMF of 0.2 T, although iron content in each osteoblast was increased, the cell proliferation did not change significantly. This inconsistent result is still a puzzle, because there is no study on changes in iron content during cell proliferation under SMFs.

Bone ALP, specifically synthesized by osteoblasts, removes phosphate group to form hydroxylapatite deposited in bone, reflecting the biosynthetic activity of osteoblast [39]. ALP activity is a sensitive and reliable indicator of osteoblast differentiation. ALP activity and mineralization function of human osteoblast cells (hFOB1.19) were decreased by ferric ammonium citrate (FAC, a complex salt composed of iron, ammonia, and citric acid) and increased by deferoxamine (DFO, an iron chelator) in a concentration-dependent manner [22, 40]. Here, we found that iron content in osteoblasts under HiMF of 16 T was decreased at day 2. Consistently, ALP activity and mineralization formation of 16 T treated osteoblasts were found to be facilitated in HiMF after continuous exposure 8 days. These results indicate that osteoblast differentiation under 16 T is promoted possibly by reducing the level of iron in cells. In addition, ALP activity and osteoblast differentiation was restrained under 500 nT with increased iron levels. However, although osteoblast differentiation was inhibited under 0.2 T, there was not found any changes in iron content of osteoblasts. Our studies demonstrate that SMF effect is highly dependent on flux intensity, and iron is potentially involved in this effect. The flux intensities used in this study were respectively discussed below.

GMF is an essential element on the earth. Conversely, elimination of GMF (i.e., HyMF) poses many adverse impacts on living organisms [1]. We previously found that HyMF of 300 nT alone did not lead to the bone loss, microstructure alterations, and mechanical properties in rat femurs, but elevated the concentrations of serum iron and aggravated bone loss induced by hindlimb unloading in rat [10]. Iron is a trace element that has important functions in vivo. In the skeletal system, both excess and insufficient iron can reduce bone mass. In vitro, iron overload inhibited osteoblast differentiation, such as the activity of ALP, the deposition of calcium, and the growth of hydroxyapatite crystals [23, 41]. In the current study, HyMF of 500 nT increased the level of iron in cells at the early stage. Afterwards, osteoblast differentiation and mineralization were inhibited in HyMF of 500 nT through restraining the activity of ALP, the deposition of calcium and the formation of mineralized nodule. During long-term space exploration, osteoporosis tends to occur especially in load-bearing bone for astronauts due to lacked gravity stress [42, 43]. Data from Mir and ISS space stations shows that mechanical stimulation in the form of exercise is still not enough to prevent bone loss in long-duration spaceflight [44, 45]. Considering the lack of GMF in outer space, this indicates that HyMF may aggravate the bone loss due to microgravity [10]. Moreover, data from the Spacelab 1 mission showed that ferritin increased 53% by the seventh day of spaceflight and 62% by landing day [46]. Iron storage and availability are increased after spaceflight [47]. Therefore, it is possible that increased tissue iron availability is the reason for spaceflight-induced bone loss.

The results demonstrated that MMF of 0.2 T decreased nodules formation, which was not consistent with the prevalent opinion. Most researches show that osteoblast differentiation can be accelerated under MMF, while several studies demonstrate the inconstant results. For example, osteoblast differentiation in MG63 cells was unaltered at 0.25 and 0.32 T, but increased at 0.4 T [48, 49]. For MC3T3-E1 cells, intensities of 150 mT [16], 250 mT [17], and 8 T [18] have a beneficial effect, while 0.2 T used in the present study decreases bone formation. Considering the diversities of intensity, duration, cell type, and treatment, it is impossible to draw a convincing conclusion that what the lowest or highest intensity is to enhance or decrease bone formation. In this study, iron content of osteoblast did not show any change after 0.2 T treatment for 2 days. However, our previous study has observed an increased iron accumulation in 10 days differentiation under 0.2 T. These seemingly contradictory findings can be attributed to the length of exposure time and the different stages of mineralization. The exact mechanisms are not clear yet. There may be an “amplitude window” around 0.2 T for the promotion of differentiation and the variation of iron in osteoblast. On account of this, it is necessary to investigate the MMF effects by using the same stimulus parameters and cell type and to find the corresponding threshold. This work will be our next research aims.

HiMF generated by superconducting technology has been widely used in medical and engineering fields, so there is a great potential for exposure to higher magnetic flux densities up to tesla order. Our studies showed that HiMF of 16 T promoted not only osteoblast proliferation, but also mineralization process. Furthermore, HiMF of 16 T increased the level of iron in cells and reduced in mediums, meanwhile the mRNA expression of TFR1 was suppressed while the expression of FPN1 was increased. These results indicate that HiMF inhibits iron uptake while promoting iron efflux in osteoblastic MC3T3-E1 cells. But in a previous study, we have shown that iron levels of osteoblast were increased with more bone nodules after 10 days differentiation in HiMF of 16 T [26]. This may be attributable to the cellular and molecular modifications induced by SMF even with the same parameters are highly dependent on the biological status of the exposed cells, such as age of the cells, mitogen activation, and so on [50, 51]. Osteoblast growth and differentiation can be characterized by three principal periods: proliferation, matrix maturation, and mineralization. Under most circumstances, the different stages are not all promoted or prohibited under a specific intensity of SMF. Imaizumi et al. [17] investigate the long-term effects of SMF on osteoblast differentiation in MC3T3-E1 cells. After 1 month of continuous exposure, ALP activity is not altered at an early phase, but mineralization process is enhanced. These findings suggest that different stages of osteoblast differentiation may have different responses to SMF stimulus.

Iron can exist in two valence states: Fe(II) and Fe(III) whose magnetic properties are quite different [52]. Fe(II) can be either paramagnetic with the effective spin 2 (high-spin state) or diamagnetid (low-spin state), while Fe(III) is all the time paramagnetic with an effective spin of 5/2 (high-spin state) or 1/2 (low-spin state). All these states depend on the ligand atoms and paramagnetic ions always interact with the magnetic field proportional to the effective spin. In cells, Fe(III) is generally present in various proteins or enzymes which participate in different biological activities of cells; Fe(II) as a crossroad of cell iron metabolism, exist in cellular labile iron pool (LIP) which is defined as a pool of redox-active iron complexes [53]. Therefore, in our next study, it is necessary to detect the changes of Fe(II) and Fe(III) under SMFs, respectively, and it would help us to understand the mechanism of SMFs affecting the iron metabolism in cells.

Several studies have been conducted to address the potential action mechanism of SMF. Cell membrane is implicated as a primary target for transmitting extracellular signals inside the cell. Under SMF, membrane phospholipids are subjected to magnetic torque due to diamagnetic anisotropy and rotate according to the direction of SMF, which deforms cell membrane, influencing its fluidity [54]. This arrangement of phospholipids may lead to changes in ion channels, and proteins embedded in membrane. Lin et al. [49] proved that SMF of 0.4 T affected membrane fluidity in osteoblastic MG63 cells. Importantly, it is well known that Tf-bound iron is transported into cells via the membrane endocytosis [55], and Fe(II) is transported into cells through the divalent metal transporter 1 (DMT1) on the membrane [56]. Nevertheless, we still do not know the exact mechanism of action that which molecules and channels are activated or disabled. Also, the relationship with direction, amplitude, time, and their combination is more complicated and not clear yet. SMF plays great roles in modulating the generation or reduction of reactive oxygen species (ROS). ROS can cause lipid peroxidation, alters cell membrane composition and fluidity, and damages proteins and DNA [57]. In addition, it was reported that ROS could inhibit differentiation of osteoblast MC3T3-E1 [58]. In our work, iron element, associated with regulating oxidative stress, was found to be altered during osteoblast differentiation. It is speculated that the decreased osteoblast mineralization under 500 nT may be due to accumulated iron. Our previous study [59] also demonstrated the modulation of cell biomechanical property under 0.2 T was accompanied by the alteration of proliferation, adhesion, cytoskeleton arrangement etc. During differentiation, the level of elastic modulus in osteoblast gradually decreases [60]. It is suggested that the response of osteoblast differentiation to SMFs may be associated with cell biomechanical property. Although many questions regarding the action mechanism remain unclear, study of the correlation between magnetic field and osteoblast activity would shed new lights that could improve our further understandings of bone health under SMFs and determine therapeutic parameters in treating or preventing human bone disorders on the Earth or in outer space.

In summary, SMFs do not have acute lethal effects on osteoblast, offering opportunities for osteoblast study in basic and application research. Osteoblast differentiation was controllable by various SMFs with different flux intensity. Moreover, iron element was altered by SMFs during osteoblast proliferation and differentiation. These results will shed new light on the corresponding mechanisms and osteoporosis treatment. From this perspective, SMF could be used as a non-invasion physical therapy to maintain health and treat disorders in bone.
